# The double-edged role of hydrogen sulfide in the pathomechanism of multiple liver diseases

**DOI:** 10.3389/fphar.2022.899859

**Published:** 2022-12-16

**Authors:** Bihan Liu, Shanshan Wang, Ming Xu, Yanan Ma, Rui Sun, Huiguo Ding, Lei Li

**Affiliations:** ^1^ Department of Hepatology and Gastroenterology, Beijing Youan Hospital, Capital Medical University, Beijing, China; ^2^ Beijing Institute of Hepatology, Beijing Youan Hospital, Capital Medical University, Beijing, China; ^3^ Brainnetome Center and National Laboratory of Pattern Recognition, Institute of Automation, Chinese Academy of Sciences, Beijing, China; ^4^ School of Artificial Intelligence, University of Chinese Academy of Sciences, Beijing, China

**Keywords:** hydrogen sulfide, liver fibrosis, liver ischemia−reperfusion injury, hepatocellular carcinoma, acute liver failure, non-alcoholic fatty liver disease

## Abstract

In mammalian systems, hydrogen sulfide (H_2_S)—one of the three known gaseous signaling molecules in mammals—has been found to have a variety of physiological functions. Existing studies have demonstrated that endogenous H_2_S is produced through enzymatic and non-enzymatic pathways. The liver is the body’s largest solid organ and is essential for H_2_S synthesis and elimination. Mounting evidence suggests H_2_S has essential roles in various aspects of liver physiological processes and pathological conditions, such as hepatic lipid metabolism, liver fibrosis, liver ischemia‒reperfusion injury, hepatocellular carcinoma, hepatotoxicity, and acute liver failure. In this review, we discuss the functions and underlying molecular mechanisms of H_2_S in multiple liver pathophysiological conditions.

## Introduction

**FIGURE 1:**
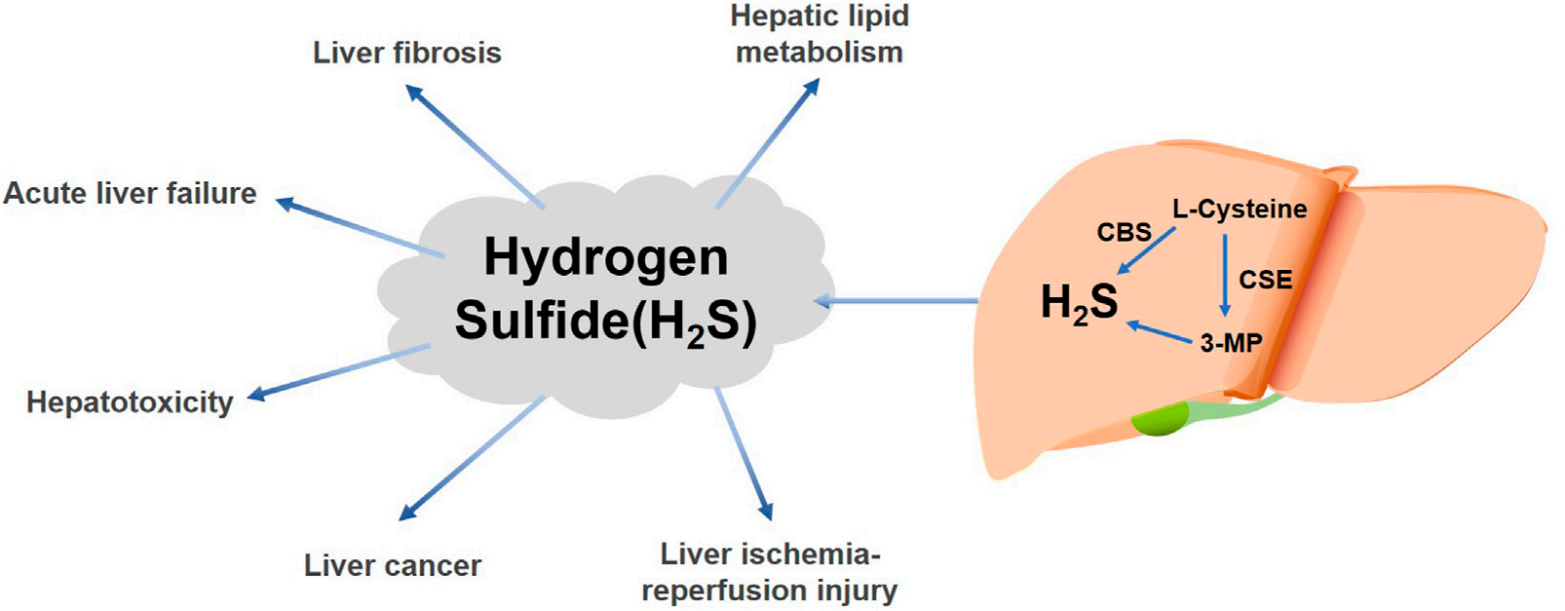
Effects of liver-derived H_2_S on various liver diseases. H_2_S is produced in the liver by the enzymatic reactions of cysteine gamma-catabolase (CSE) (Jia et al., 2022), cystathionine β-synthase (CBS) (Roy et al., 2012; Zuhra et al., 2020a), and 3-mercaptopyruvate (3-MP) *via* L-cysteine (Roy et al., 2012; Abdollahi Govar et al., 2020; Dilek et al., 2020). H_2_S can have different effects in various liver diseases.

Hydrogen sulfide (H_2_S) is one of the three recognized gaseous signaling molecules in mammals (Yang et al., 2019b). Existing studies have demonstrated that endogenous H_2_S is created in mammalian systems by enzymatic and non-enzymatic mechanisms (Yang et al., 2019b; Loiselle et al., 2020). The enzymatic delivery pathways in mammalian cells and tissues are those related to cystathionine gamma-lyase (CSE) (Jia et al., 2022), cystathionine β-synthase (CBS) (Roy et al., 2012; Zuhra et al., 2020a), and 3-mercaptopyruvate sulfotransferase (3-MST) (Roy et al., 2012; Abdollahi Govar et al., 2020; Dilek et al., 2020) (Figure 1). Moreover, some microorganisms located in the intestine are also capable of producing H_2_S (Loiselle et al., 2020; Scammahorn et al., 2021).

Furthermore, hepatic stellate cells can boost H_2_S production (Ma et al., 2022). Mounting evidence shows that H_2_S plays a critical role in a variety of physiological and pathological processes, including the respiratory, cardiovascular, gastrointestinal, and central nervous systems, as well as in the kidneys (Shackelford et al., 2021) and in the inflammatory response and antioxidant defense systems (Shi et al., 2019).

The liver is the body’s largest organ, and it plays an important role in lipid, glucose, and xenobiotic metabolism and in resistance to oxidative stress and defense against invading microorganisms (Norris et al., 2011; Andrade et al., 2019; Chen et al., 2019). It is also important for H_2_S synthesis and removal (Dilek et al., 2020). Moreover, CSE, CBS, and 3-MST, which are responsible for H_2_S production to varying degrees, have been found in the liver (Bełtowski, 2019). H_2_S metabolism in the liver is linked to insulin sensitivity, glucose metabolism, lipoprotein production, and mitochondrial bioenergetics and biogenesis (Sun et al., 2021). Most importantly, H_2_S synthesis and signal transmission in the liver are disrupted in a variety of liver disorders, such as non-alcoholic fatty liver disease (NAFLD), liver fibrosis, hepatic ischemia/reperfusion (I/R) injury, and liver cancer (Loiselle et al., 2020).

In recent decades, studies have focused on the importance of H_2_S in liver growth and development, and the relationship between H_2_S and both liver functions and diseases have partially been explored; thus, the underlying mechanisms of H_2_S-mediated hepatoprotection or injury have been gradually revealed. For this article, we retrospectively reviewed recent studies of H_2_S in various liver diseases and found that H_2_S not only has a positive protective effect against liver disease but also has a non-negligible pathogenic role in certain liver diseases. In particular, we highlight the mechanisms by which H_2_S is involved in these metabolic processes and relevant therapeutic targets.

## The effects of H_2_S in non-alcoholic fatty liver disease

H_2_S acts as an essential novel regulator in lipid metabolism and plays a role in many diseases, such as diabetes, obesity, and cardiovascular disorders (Wu et al., 2019; Corvino et al., 2021; Zhao et al., 2022). The liver is an important organ for lipid metabolism in the body and is responsible for the accumulation, storage, and consumption of lipids (Stefan et al., 2019). Researchers have recently given special attention to H_2_S and the control of lipid metabolism in the liver—a relationship which has important consequences for the progression of liver disease. Non-alcoholic fatty liver disease (NAFLD) is a broad class of liver diseases that range from steatosis to the more severe form of non-alcoholic steatohepatitis (NASH), a condition that can aggravate liver fibrosis and liver cirrhosis (Abd El-Kader and El-Den Ashmawy, 2015). NASH is now considered a common chronic liver disease that is present in 25% of the global population (Wu et al., 2020).

The involvement of H_2_S in liver health has been explored in many studies. A previous study on NAFLD showed that endogenous H_2_S levels in hepatocytes of oleic acid-treated mice were lower than the levels in an untreated group. Next, the authors investigated the effect of exogenous H_2_S on cell growth in the oleic acid group. The results showed that H_2_S slowed liver lipid deposition through the activation of farnesol X receptors and increased the proliferation and survival of oleic acid-treated cells (Ruan et al., 2019; Loiselle et al., 2020; Xu et al., 2022). In addition, oleic acid treatment resulted in the arrest of hepatocytes in the G1 phase of the cell cycle—a process which was then reversed by H_2_S (Loiselle et al., 2020; Xu et al., 2022). Related experimental results demonstrated that H_2_S could promote autophagy and inhibit apoptosis in human hepatocytes through the ROS/PI3K/AKT/mTOR pathway that is mediated by reactive oxygen species (ROS), thereby alleviating the high-fat diet (HFD)-induced NAFLD (Wu et al., 2020). This research shows that H_2_S could be applied to treat NAFLD. It is also worth noting that exogenous NaHS can alleviate lipid accumulation and that the 3-MST knockdown has been shown to significantly improve hepatic steatosis in high-fat diet-fed mice (Li et al., 2018). Therefore, further research is needed to fully understand the impact of H_2_S on NAFLD.

However, *in vitro* systems cannot accurately simulate the natural *in vivo* physiological environment; therefore, research has increasingly focused on *in vivo* models, which are believed to provide various types of vital evidence for the relationship between H_2_S and lipid metabolism that cannot be realized *in vitro*. Various knockout (KO) animal models, including CBS-KO, CSE-KO, and 3-MST-KO mouse models, have revealed critical roles of H_2_S-producing enzymes in hepatic lipid metabolism (Loiselle et al., 2020).

A study that used a rat fatty liver ischemia/reperfusion injury (IRI) model revealed that the homozygous CBS mutant mice died within 5 weeks of birth, and further histological examination revealed an enlarged and lipid droplet-filled liver (Zhang et al., 2012). Similar results were shown in another study. Compared to the control group, the CBS-deficient mice had more lipid accumulation in the liver (Li et al., 2018). These results suggested that fatty acids reduced endogenous H_2_S levels by inhibiting the CSE-dependent pathway in the liver, which would promote fat accumulation and subsequently lead to NAFLD (Mani et al., 2015). S Mani noted that, in comparison to the wild-type mice, the CSE-KO mice had much higher cholesterol levels in the plasma and liver when fed a HFD. Dyslipidemia, microvascular fat accumulation, changes in liver pigments, and hepatic damage were all observed in the mice. Finally, the CSE-KO mice that were fed a HFD developed a fatty liver (Roehlen et al., 2020).

In conclusion, the effects and mechanisms of H_2_S in hepatic lipid metabolism have become increasingly clear. H_2_S and its synthase have important protective effects on hepatic lipid metabolism, significantly improving hepatic lipid deposition. Otherwise, the level of H_2_S is reduced, which increases lipid deposition and promotes the development of NAFLD. To date, the precise mechanism and clinical significance of H_2_S and its synthase have yet to be elucidated.

## The effects of H_2_S in liver fibrosis

In developed countries, death from fibrotic diseases including chronic kidney disease, liver cirrhosis, idiopathic pulmonary fibrosis, and chronic disease accounts for 45% of human mortality, posing a serious danger to health worldwide (Zou et al., 2009; Song et al., 2015; Ni et al., 2018). Liver fibrosis accounts for a large proportion of these fibrotic diseases. The persistent buildup of extracellular matrix (ECM) over type I collagen is the primary cause of liver fibrosis (Lambrecht et al., 2015). Many molecules are involved in its development, with inflammation and oxidative stress being well-known regulatory targets. In many chronic liver illnesses, such as viral hepatitis, NASH, and NAFLD, liver fibrosis is an unavoidable pathological process (Song et al., 2015). A growing number of studies are demonstrating that the suppression of endogenous H_2_S might be related to the progression of fibrosis in humans and that H_2_S supplementation has protective and therapeutic effects against fibroproliferative diseases and syndromes of common organs (liver, lung, kidney, and heart), mainly due to its anti-inflammatory, antioxidant, and antifibrotic effects (Kabil et al., 2014; Pant et al., 2016; Bai et al., 2019).

H_2_S may affect hepatic fibrosis development in two ways (Mohammed et al., 2021): 1) H_2_S has been shown to act as an antifibrotic molecule by significantly reducing the levels of TNF-α, IL-1β, IL-6, and soluble intercellular adhesion molecule (ICAM)-1 in the rat serum to suppress the inflammatory response (Tan et al., 2011; Mohammed et al., 2021) and 2) it exerts antioxidant effects by increasing the activity and expression of the catalase, copper−zinc superoxide dismutase and manganese superoxide dismutase, thereby effectively inhibiting the progression of fibrosis (Jung et al., 2013). An *in vivo* study found that S-allyl-cysteine (SAC), an endogenous provider of H_2_S, could relieve CCl4-induced liver fibrosis in rats by inhibiting the STAT3/SMAD3 pathway (Gong et al., 2018). Further experimental studies have shown that SAC treatment reduces the expression of both inflammatory factors and fibrogenic cytokines and increases the expression of antioxidant enzymes (Kodai et al., 2007). H_2_S has been shown to induce cell cycle arrest, apoptosis, and vasodilation by activating p53, p21, caspase-3, and MMP-2, by promoting their overexpression and by downregulating the Bcl-xL expression (Ma et al., 2018). H_2_S also ameliorates liver fibrosis through its anti-inflammatory and antioxidant properties, thereby alleviating portal hypertension (Zou et al., 2009; Gong et al., 2018; Ma et al., 2018; Damba et al., 2019; Ali et al., 2020).

However, there are several factors associated with H_2_S that contribute to the formation of liver fibrosis. A perspective, taking this aspect into account was proposed by C.G. Zou et al., who found that the precursor of H_2_S synthesis, homocysteine, enhances the activation of human hepatic stellate cells by activating the PI3K/Akt signaling pathway (Ali et al., 2020). The T. Damba team further verified this hypothesis. They found that both endogenous and exogenous H_2_S can increase the proliferation and activation of hepatic stellate cells by increasing the glycolysis extracellular acidification rate (ECAR) and the oxygen consumption rate (OCR) of mitochondrial oxidative phosphorylation, thus promoting the metabolic activity of hepatic stellate cells (Jiménez-Castro et al., 2019) and further promoting liver fibrosis formation.

These various reports show that additional studies investigating the importance of H_2_S in liver fibrosis and H_2_S chemical pathways are necessary and that future works should include confirmation with animal experiments and cellular studies.

## The effects of H_2_S on liver ischemia‒reperfusion injury

Ischemia‒reperfusion (I/R) is a well-known pathological condition marked by a temporary decrease in blood supply to an organ or region, followed by vascular recovery and downstream tissue damage (Yang et al., 2018b). It is a consequence of hemorrhagic shock and resuscitation, trauma, liver resections, liver transplantation, bowel infarction, and, especially, liver failure. Hepatic I/R damage has become a global health issue (Nastos et al., 2014). Various clinical experiments and basic studies point to diverse molecular mechanisms in this process, including those related to neutrophils and liver Kupffer cells, proinflammatory cytokines, adhesion molecules, mitochondria, oxidative stress, anaerobic metabolism, and intracellular calcium overload (Kang et al., 2009; Zhai et al., 2013). Thus, novel drugs that have anti-oxidative, anti-inflammatory, and cytoprotective effects may protect the liver from I/R injury (Krylatov et al., 2021). Currently, H_2_S is known to be critical in the treatment of liver I/R injury (Wu et al., 2015). Due to the thorough investigation of H_2_S and liver I/R injury, the mechanism by which H_2_S protects against I/R injury has begun to be elucidated.

There have been significant advances in animal studies focusing on the molecular pathways of H_2_S in I/R injury (Lu et al., 2018; Fu et al., 2019; Ibrahim et al., 2021). Recent studies have found that fatty livers are more susceptible to ischemia/reperfusion (I/R) damage during liver surgery and transplantation (Varela et al., 2011). In a rat fatty liver IRI model, the influence of H_2_S on IRI was thoroughly investigated. According to the findings, H_2_S mitigated changes in liver pathology and lowered the levels of aspartate aminotransferase (AST), alanine aminotransferase (ALT), and malondialdehyde (MDA). Moreover, H_2_S decreased oxidative stress levels and the expression of inflammatory factors and slowed the apoptosis of hepatocytes (Younis et al., 2016). In addition, treatment with silymarin protected against hepatic I/R in insulin-resistant rats through anti-inflammatory, antioxidant, and anti-apoptotic effects and the inhibition of H_2_S synthesis (Lu et al., 2018; Liu et al., 2020). Therefore, retaining an appropriate level of H_2_S in ischemia‒reperfusion (I/R) is imperative in protecting the liver from injury. NaHS protects the liver against I/R and, as a donor of H_2_S, is protective against hepatic I/R injury, a process associated with the activation of antioxidant enzymes and decreased expression of hepatic tumor necrosis factor-α (TNF-α), MDA, and caspase-3 (Fu et al., 2019).

Cold ischemia‒reperfusion injury (IRI) poses a significant threat to the success of solid organ transplantation (SOT) (Muller et al., 2022). A study discussing the molecular mechanisms underlying the role of H_2_S donor molecules in liver transplantation showed that H_2_S could significantly attenuate IRI during liver transplantation by inhibiting a range of interrelated cells and molecules, including those related to microcirculatory dysfunction and microvascular dysfunction, mitochondrial damage, inflammatory responses, cellular injury, cell death, and other destructive molecular pathways, while promoting the protective pathways (Balaban et al., 2011; Fu et al., 2019). These promising findings will be the basis of the clinical application of H_2_S in the future (Dugbartey et al., 2021).

In addition, another piece of evidence from an experimental model of organ transplantation in mice suggests that exogenous administration of H_2_S donor molecules during graft preservation significantly improves liver microcirculation, morphology, and function. Moreover, a significant increase in liver antioxidant enzyme levels and activity was also observed. In contrast, lactate dehydrogenase, malondialdehyde (an indicator of lipid peroxidation byproducts and ROS production), and other markers of liver injury were significantly reduced. These new findings suggest that adding H_2_S donor molecules during liver transplantation can play an important role in significantly increasing the survival rate of transplanted organs, mitigating liver IRI injury during transplantation, reducing complications, and improving patient prognosis (Balaban et al., 2015).

These findings imply that focusing on H_2_S could be a promising new strategy for combating I/R-induced liver damage. The molecular targets of H_2_S in liver I/R damage, however, are still unknown. Since different doses of H_2_S generated by the donor may have varying therapeutic effects, the optimum dose range should be investigated further for improved efficacy.

## The effect of H_2_S in hepatocellular carcinoma

Liver cancer is the world’s sixth most prevalent malignancy and the fourth leading cause of cancer-related death (Yang et al., 2019c; Li et al., 2021b). Among liver cancers, the most common kind is hepatocellular carcinoma (HCC). The primary risk factors for HCC include viral infection, chronic alcohol consumption, and obesity-related NASH. HCC pathophysiology is a complex multistep process (Cao et al., 2019). Currently, HCC continues to be a global health threat, with morbidity and mortality rates increasing dramatically. As a consequence, the monitoring of HCC and early detection are regarded as vital methods to improve the effectiveness of treatment (Malik et al., 2020). Studies have revealed that angiogenesis and immune evasion are key core issues in the tumor microenvironment for liver cancer progression and treatment failure (Motz and Coukos, 2011; Fousek et al., 2021; Pinter et al., 2021; Zhou et al., 2021). In a complex tumor microenvironment composed of hepatocytes, liver sinusoidal endothelial cells (LSECs), hepatic stellate cells (HSCs), immune cells, and extracellular matrix, the development of HCC is closely related to the infiltration of immune cells and immune evasion in the tumor microenvironment (TME) (Muñoz et al., 2021). Increasing evidence has shown that H_2_S plays a key role in the occurrence and development of HCC. Recently, CBS was found to inhibit Treg cell infiltration and induce apoptosis in human HCC cells by suppressing the PRRX2/IL-6/STAT3 signaling pathway. CBS deficiency promoted Treg-mediated immune evasion and tumor growth in mice, suggesting that the CBS/H_2_S axis may control immune evasion in the TME (Xu et al., 2021; Zhou et al., 2021). The overexpression and overactivation of the immunosuppressive enzyme indoleamine 2,3-dioxygenase 1 (IDO1) is a key mechanism of immune escape from cancer. In a mouse liver cancer model, exogenous H_2_S inhibited IDO1 expression by blocking the STAT3 and NF-κB pathways and reduced IDO1 activity through the H_2_S/NO crosstalk, effectively stopping tumor progression in mice (Yang et al., 2019a). Furthermore, the H_2_S donor effectively suppresses tumor development in mice with hepatocellular carcinoma models (Ngowi et al., 2021). These findings suggest that targeting the CBS/H_2_S axis might be a novel method for therapeutic immunotherapy in HCC.

In addition to immunotherapy, H_2_S can also promote apoptosis of HCC cells directly (Wang et al., 2017). Our previous research revealed that exogenous H_2_S could induce HCC cell autophagy and further promote apoptosis by inhibiting the PI3K/AKT signaling pathway (Wang et al., 2017). In the TME, hepatic stellate cells activate the JNK/JunB signaling pathway through the release of H_2_S, upregulate the expression of TNFSF14, and promote apoptosis in hepatocellular carcinoma cells (Ma et al., 2022). A similar result was found for endogenous H_2_S; Zhou et al. found that the activation of the CBS/H_2_S axis in HCC cells upregulated the expression of cleaved caspase-3 and promoted HCC cell apoptosis (Sakuma et al., 2019).

In addition, H_2_S donors are able to regulate apoptosis by interacting with signaling pathways related to apoptosis in cancer cells (Szadvari et al., 2019). Treatment with GYY4137, an H_2_S donor, increased the expression of caspase-9, a marker of apoptosis, in human HCC cells (Zhao et al., 2015). Moreover, treatment with NaHS suppressed the anti-apoptotic markers in B-cell lymphoma 2 (Bcl-2) by regulating the p53 and p38 MAPK pathways (Pan et al., 2014; Zhao et al., 2015), thus promoting cancer cell apoptosis (Zuhra et al., 2020b; Xia et al., 2021). Moreover, H_2_S acts as a stimulator of mitochondrial electron transfer, and endogenous H_2_S stimulates adenosine triphosphate (ATP) production in cancer cells, which plays an important role in preventing mitochondrial fission and maintaining mitochondrial DNA repair (Fortibui et al., 2021).

There is evidence that metabolic reprogramming of cancer is a determinant in the anticancer-related immune response (Zhang et al., 2020). For example, sulfur-related metabolism is still a novel direction of research in human HCC tumor tissues. Recent studies that focused on gene expression profiling in HCC *via* Gene Set Enrichment Analysis (GSEA) revealed that sulfur amino acid metabolism in HCC was downregulated. Cell viability experiments showed that H_2_S had notable anticancer effects in human HCC cells. H_2_S can also provide sulfane sulfur, which mediates reactive sulfur species (RSS)-induced anti-HCC effects in tumor cells. Finally, it was shown that sulfur metabolism in HCC had been reprogrammed and a potential therapeutic strategy for HCC was proposed (Wang et al., 2020b). Acetaldehyde dehydrogenase (ALDH) is the primary enzyme in the liver that regulates acetaldehyde metabolism (Toledo-Guzmán et al., 2019). It has been shown that ALDH can alter a variety of biological features in cancer stem cells and could be used as a cancer stem cell diagnostic marker (Duan et al., 2016; Iciek et al., 2018). Evidence suggests that ALDH plays a significant role in cancer recurrence (Yang et al., 2018a). Based on these findings, it is possible that ALDH is regulated by sulfur substances, which inhibit its enzymatic activity, thus making cancer cells more sensitive to conservative treatment.

In addition, *in vitro* and *in vivo*, a combination of kelp and *Curcuma zedoaria* inhibited the growth and metastasis of liver cancer cells by reducing the generation of endogenous H_2_S and regulating the pSTAT3/BCL-2 and VEGF pathways, according to a prior study (Han et al., 2019). Another mechanistic study found that the inhibition of reactive oxygen species (ROS), the activation of the STAT3/Akt/Bcl-2 pathway, and the induced metastatic capacity of HCC cells were the leading causes of enhanced drug resistance in HCC (Wang et al., 2018). Studies have also found that human HCC cells with high CBS expression had low sensitivity to sunitinib and doxorubicin (DOX), while the knockdown of CBS markedly increased the sensitivity of HCC fineness to DOX and sunitinib. Therefore (Wang et al., 2018), it was concluded that CBS overexpression conferred resistance to HCC cells (Stravitz and Lee, 2019).

**FIGURE 2:**
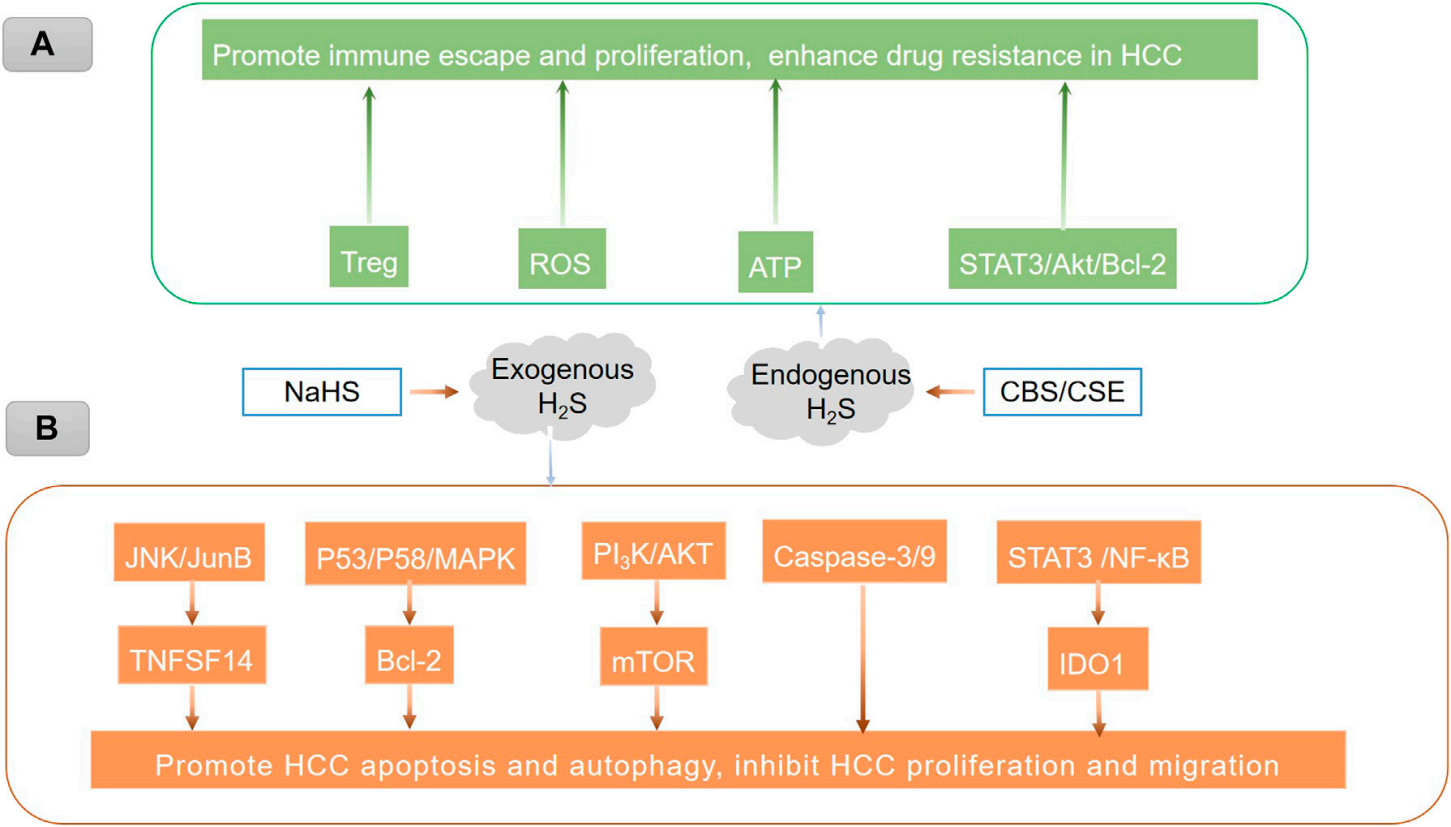
Endogenous and exogenous H_2_S play different roles in HCC through different mechanisms. **(A)** Endogenous H_2_S promotes immune escape and proliferation and enhances drug resistance in HCC, which may be related to the promotion of Treg-mediated immune evasion (Xu et al., 2021; Zhou et al., 2021), the stimulation of ATP production (Fortibui et al., 2021), and the activation of the STAT3/Akt/Bcl-2 pathway (Wang et al., 2018). The inhibition of reactive oxygen species (ROS) (Wang et al., 2018). **(B)** Treatment of hepatoma cells with exogenous H_2_S could induce HCC cell autophagy by activating the JNK/JunB/TNFSF14 signaling pathway (Ma et al., 2022) and the STAT3/Akt/Bcl-2 pathway (Han et al., 2019), upregulating caspase-3/9 (Sakuma et al., 2019), inhibiting the PI3K/AKT/mTOR signaling pathway (Wang et al., 2017), regulating the p53/p58/MAPK pathway (Zhao et al., 2015), blocking the STAT3/NF-κB/IDO1 pathway, and suppressing anti-apoptotic markers, thus promoting hepatoma cell apoptosis.

These findings imply that H_2_S has contradictory effects on HCC. Explicitly speaking, exogenous H_2_S can cause cancer cell death, while endogenous H_2_S can promote cancer. These results indicate that supplementation and restraint of H_2_S production are two different ways to treat cancer. As a result, H_2_S is expected to play dual roles in the development of HCC (Wu et al., 2017). In the future, it is critical to investigate the mechanism of H_2_S in HCC in greater depth (Figure 2).

## The effects of H_2_S on acute liver failure

Acute liver failure (ALF) is an uncommon and serious complication related to sudden hepatic injury that lasts several days or weeks and is marked by rapid liver destruction, multi-organ failure, and a high death rate (Sen, 2017; Kolodziejczyk et al., 2020). Paracetamol poisoning, hepatic ischemia, viral and autoimmune hepatitis, drug-induced liver injury from prescription medicines, and herbal and nutritional supplements are all potential causes of abrupt liver failure (Kumar and Sandhir, 2018; Yuan et al., 2020). H_2_S has been shown to ameliorate liver complications in previous investigations (Dong et al., 2020; Kožich and Stabler, 2020). A recent study explored the possibility of H_2_S having a protective impact in ALF. The addition of sodium thiosulfate (STS), an H_2_S donor, effectively alleviated D-galactosamine (GalN)- and lipopolysaccharide (LPS)-induced acute liver failure in the wild-type mice by activating Akt- and Nrf2-dependent signaling and by inhibiting GalN/LPS-induced JNK phosphorylation. This suggests that the inhibition of CSE or that the introduction of STS can prevent acute inflammatory liver failure by increasing thiosulfate levels and upregulating antioxidant and anti-apoptotic defenses in the liver (Shirozu et al., 2014). In another study, a thioacetamide (TAA)-induced ALF mouse model was used, and the results showed that NaHS treatment reduced cognitive deficits, enabled the retention of TAA-induced spatial orientation learning, and reduced serum AST and ALT levels and ammonia concentrations in mice (Milewski et al., 2021). These findings suggest the therapeutic potential of H_2_S to reduce cognitive deficits and liver dysfunction in ALF mice but the exact biological mechanism remains to be explored (Abo El Gheit et al., 2020; Milewski et al., 2021). Compared to the wild-type mice, the CSE-deficient mice showed a significant attenuation of burn-induced elevations in circulating alkaline aminotransferase and blood urea nitrogen and creatinine levels, suggesting that CSE deficiency has a protective effect against burn-induced impairment of the liver and kidney function. Plasma levels of several burn-induced inflammatory mediators (TNF-α, IL-1β, IL-4, IL-6, IL-10, and IL-12) were significantly lower in the CSE-deficient mice after a burn injury than in the plasma from the wild-type controls. In conclusion, in a mouse burn model, the absence of CSE improved organ function, attenuated the inflammatory response, and effectively limited the progression of multi-organ failure. However, the exact mechanism remains to be explored (Ahmad et al., 2017).

In a study of burn-induced acute liver injury in mice, plasma H_2_S levels and H_2_S synthesis activity were significantly increased in the liver after a burn injury, and NaHS injections at the time of burn injury also led to a substantial increase in liver myeloperoxidase (MPO) activity and a significant increase in the systemic inflammatory response, inducing multi-organ damage, including liver injury. This suggests that H_2_S can significantly exacerbate burn-induced acute liver injury (Zhang et al., 2010).

These findings imply that H_2_S may play a double-edged role in acute liver failure, and more in-depth studies are still needed to validate it further.

## The effect of H_2_S on acute liver pathology

Liver toxicity refers to the damage to the liver produced by a variety of prescription and over-the-counter medications, including natural medicines, biologics, dietary supplements, nutraceuticals, and some traditional Chinese medicines (Jaeschke, 2015). Drug-induced liver damage is a rare but serious medical issue (Leise et al., 2014; Wang et al., 2020a). In a limited number of patients, the use of multiple drugs has caused serious liver injury and acute liver failure (<1:10,000) (Chalasani et al., 2008). Although its incidence in the population is very low, the high likelihood of acute liver failure in patients with acute liver injury still requires much attention.

A liver toxicity study in mice found that H_2_S significantly inhibited oxidative stress, inflammation, and apoptosis induced by polystyrene microplastics (mic-PS). H_2_S increased the expression of NAD(P)H:quinone oxidoreductase 1 (NQOl) and heme oxygenase-1 (HO-1) by promoting the nuclear accumulation of the nuclear factor-E2-related factor (Nrf2), thereby reducing the apoptotic and inflammatory responses induced by mic-PS in mouse hepatocytes. This revealed the hepatic toxic effect of mic-PS and the protective effect of H_2_S on mic-PS-induced liver injury (Li et al., 2021a). In a study of acetaminophen (APAP)-induced hepatotoxicity in mice, treatment with H_2_S significantly reduced serum levels of AST, ALT, IL-33, and TNF-α. It attenuated APAP-induced hepatocyte apoptosis in mice by inhibiting the JNK/MAPK signaling pathway, thus effectively reducing APAP-induced hepatotoxicity (Li et al., 2019; Saleh et al., 2021). In addition, another hepatotoxicity study in rats demonstrated that H_2_S protects the liver in methotrexate (MTX)-stimulated rats by acting as anti-inflammatory, antioxidant, and anti-apoptotic agent functions, which are most likely mediated by H_2_S through the modulation of the IL-6/STAT3 pathway, initiation of the KATP pathway, and activation of endothelial nitric oxide synthase (eNOS) and transient receptor potential vanilloid 1 (TRPV1) (Fouad et al., 2020). Thus, H_2_S has a potential value for treating hepatotoxicity.

## Conclusion and perspective

In this review, we summarized and discussed the effects and potential mechanisms of H_2_S in the process of liver disease, including NAFLD, liver I/R injury, liver fibrosis, acute liver failure, liver toxicity, and hepatocellular carcinoma. Based on the research results, we found that endogenous and exogenous high and low concentrations of H_2_S may exert different effects by regulating different signaling pathways.

The effects of H_2_S on NAFLD and liver I/R injury are relatively clear. Under NAFLD conditions, both endogenous and exogenous H_2_S were able to reduce lipid deposition to inhibit the progression of NAFLD, indicating that H_2_S plays a protective role in pathological conditions in the liver. Moreover, H_2_S alleviated liver I/R injury by reducing the inflammatory reaction. However, H_2_S may act as a negative molecule in promoting the progression of liver fibrosis. Both endogenous and exogenous H_2_S could activate HSCs to increase the secretion of extracellular matrix, which participates in liver fibrosis. Beyond its roles in NAFLD and liver fibrosis, the roles of H_2_S in other liver diseases remain controversial.

Based on the findings of these studies, we determined that H_2_S plays a double-edged role in liver diseases. We suppose that there may be a certain balance between the protective and pathogenic effects of H_2_S in different liver conditions. Since there is no definite evidence to prove the existence of this balance at present, more studies are needed in the future. Considering the controversial effects of H_2_S in different liver diseases, inhibiting the synthesis of endogenous H_2_S or providing exogenous H_2_S can effectively alleviate the progression of diseases.

Currently, H_2_S has been studied as a drug in the cardiovascular field. However, the dual role of H_2_S in different diseases should be considered when using H_2_S to treat liver diseases. Inhibition of endogenous H_2_S synthesis or the administration of exogenous H_2_S can play a positive role in the treatment of liver diseases and are, therefore, promising treatment strategies. Therefore, a better understanding of the dual role of H_2_S will provide a strong experimental basis for the treatment of different diseases and for drug research.

## Glossary

ALDH: acetaldehyde dehydrogenase
ALF: acute liver failure
ALT: alanine aminotransferase
APAP: acetaminophen
AST: aspartate aminotransferase
ATP: adenosine triphosphate
Bcl-2: B-cell lymphoma 2
CBS: cystathionine beta-synthase
CSE: cystathionine gamma-lyase
DOX: doxorubicin
ECAR: glycolysis extracellular acidification rate
ECM: extracellular matrix
eNOS: endothelial nitric oxide synthase
GalN: D-galactosamine
H_2_S: hydrogen sulfide
HCC: hepatocellular carcinoma
HFD: high-fat diet
HO-1: heme oxygenase-1
HSC: hepatic stellate cells
ICAM: intercellular adhesion molecule
IDO1: indoleamine 2,3-dioxygenase 1
IRI: ischemia/reperfusion injury
KO: knockout
LPS: lipopolysaccharide
LSEC: liver sinusoidal endothelial cell
MDA: malondialdehyde
mic-PS: microplastics
MPO: myeloperoxidase
MTX: methotrexate
NAFLD: non-alcoholic fatty liver disease
NASH: non-alcoholic steatohepatitis
NQO1: NAD(P)H:quinone oxidoreductase 1
Nrf2: nuclear factor-E2-related factor
OCR: oxygen consumption rate
ROS: reactive oxygen species
RSS: reactive sulfur species
SAC: S-allyl-cysteine
SOT: solid organ transplantation
STS: sodium thiosulfate
TAA: thioacetamide
TME: tumor microenvironment
TNF-α: tumor necrosis factor-α
TRPV1: transient receptor potential vanilloid 1
3-MST: 3-mercaptopyruvate sulfotransferase
